# Analytical and Clinical Assessment of a Portable, Isothermal Recombinase Polymerase Amplification (RPA) Assay for the Molecular Diagnosis of Urogenital Schistosomiasis

**DOI:** 10.3390/molecules25184175

**Published:** 2020-09-11

**Authors:** John Archer, Rebecca Barksby, Tom Pennance, Penelope Rostron, Faki Bakar, Stefanie Knopp, Fiona Allan, Fatma Kabole, Said M. Ali, Shaali M. Ame, David Rollinson, Bonnie L. Webster

**Affiliations:** 1 Wolfson Wellcome Biomedical Laboratories, Department of Life Sciences, Natural History Museum, Cromwell Road, London SW7 5BD, UK; j.archer@NHM.ac.uk (J.A.); T.Pennance@nhm.ac.uk (T.P.); penny.rostron@hotmail.com (P.R.); s.knopp@swisstph.ch (S.K.); f.allan@nhm.ac.uk (F.A.); d.rollinson@nhm.ac.uk (D.R.); 2 London Centre for Neglected Tropical Disease Research (LCNTDR), London W21 PG, UK; 3 Faculty of Infectious and Tropical Diseases, London School of Hygiene and Tropical Medicine, Keppel Street, London WC1E 7HT, UK; rebecca.barksby@lancet.com; 4 School of Biosciences, Cardiff University, Cardiff CF10 3AX, UK; 5 Public Health Laboratory—Ivo de Carneri, P.O. Box 122, Chake-Chake, Pemba, Tanzania; fakibakar49@gmail.com (F.B.); saidmali2003@yahoo.com (S.M.A.); shaaliame@yahoo.com (S.M.A.); 6 Swiss Tropical and Public Health Institute, Socinstrasse 57, 4002 Basel, Switzerland; 7 University of Basel, Petersplatz 1, 4003 Basel, Switzerland; 8 Zanzibar Neglected Diseases Programme, Ministry of Health, P.O. Box 236, Zanzibar Town, Unguja, Tanzania; fatmaepi@yahoo.co.uk

**Keywords:** urogenital schistosomiasis, *Schistosoma haematobium* (*S. haematobium*), point-of-care, diagnosis, recombinase polymerase amplification (RPA), control, surveillance, elimination

## Abstract

Accurate diagnosis of urogenital schistosomiasis is crucial for disease surveillance and control. Routine diagnostic methods, however, lack sensitivity when assessing patients with low levels of infection still able to maintain pathogen transmission. Therefore, there is a need for highly sensitive diagnostic tools that can be used at the point-of-care in endemic areas. Recombinase polymerase amplification (RPA) is a rapid and sensitive diagnostic tool that has been used to diagnose several pathogens at the point-of-care. Here, the analytical performance of a previously developed RPA assay (RT-ShDra1-RPA) targeting the *Schistosoma haematobium* Dra1 genomic region was assessed using commercially synthesised *S. haematobium* Dra1 copies and laboratory-prepared samples spiked with *S. haematobium* eggs. Clinical performance was also assessed by comparing diagnostic outcomes with that of a reference diagnostic standard, urine-egg microscopy. The RT-ShDra1-RPA was able to detect 1 × 10^1^ copies of commercially synthesised Dra1 DNA as well as one *S. haematobium* egg within laboratory-spiked ddH_2_O samples. When compared with urine-egg microscopy, the overall sensitivity and specificity of the RT-ShDra1-RPA assay was 93.7% (±88.7–96.9) and 100% (±69.1–100), respectively. Positive and negative predictive values were 100% (±97.5–100) and 50% (±27.2–72.8), respectively. The RT-ShDra1-RPA therefore shows promise as a rapid and highly sensitive diagnostic tool able to diagnose urogenital schistosomiasis at the point-of-care.

## 1. Introduction

Schistosomiasis is a debilitating neglected tropical disease (NTD), caused by infection with parasitic blood-fluke trematodes of the genus *Schistosoma*, that afflicts over 230 million people within 78 countries [1,2]. It is estimated that more than 85% of all global cases occur within sub-Saharan Africa, of which approximately two-thirds are caused by *Schistosoma haematobium*, the causative parasite of urogenital schistosomiasis [3,4].

Pathologies associated with urogenital schistosomiasis occur primarily as a result of the copious number of eggs produced by female adult worms which inhabit the venous plexus of the bladder [5]. Rather than being passed in the urine, to perpetuate the parasite’s life cycle, a large proportion of eggs will become sequestered throughout the genital tract. This evokes a T helper type-2 (Th2) cell-driven granulomatous response that can cause a range of clinical morbidities including severe abdominal pain, destruction of the bladder wall, haematuria, severe kidney disease and, in chronic cases, bladder cancer [1,6]. Clinical manifestations specific to women are collectively termed female genital schistosomiasis (FGS) and can include vaginal lesions, intermenstrual and post-coital bleeding, ectopic pregnancy and miscarriage [7,8]. In addition, FGS-associated destruction of the cervicovaginal mucosa is now also recognised as an important contributor to the transmission of sexually transmitted bacterial and viral infections (STIs), including human immunodeficiency virus (HIV) and human papillomavirus (HPV), which can cause cervical cancer [9,10,11].

Efforts to reduce both the transmission of schistosomes and morbidity associated with schistosomiasis rely primarily on preventative chemotherapy (PC) through mass drug administration (MDA) of the anthelmintic drug, praziquantel [12,13,14,15]. In adopting this strategy, significant gains have been made in reducing the overall burden of disease throughout many areas of sub-Saharan Africa [16,17]. As the number of individuals infected, as well as the intensity of infection within those infected individuals, is diminished, however, a sharp decline in transurinal egg output causes great difficulty in reliably detecting individuals with low levels of infection using standard diagnostic methods—urine-egg microscopy and haematuria-detecting lateral-flow strips [18,19,20,21].

Schistosomes reproduce asexually within specific freshwater snail intermediate hosts and so many cercariae can be produced after snails become infected with just a few miracidia (hatched from eggs). As such, just one human harbouring even a low level of infection, which was not entirely cleared following treatment, is sufficient to re-infect a population of freshwater snails and therefore the human population post-MDA [1,22,23,24]. As control programmes progress, reliable and robust diagnostic tools which can be easily and rapidly carried out at the point-of-care and are able to detect low levels of infection within individuals able to maintain pathogen transmission are therefore needed to achieve and sustain elimination [20,25,26,27,28,29].

Although a range of promising *Schistosoma* antigen-detecting and anti-*Schistosoma* antibody-detecting immunodiagnostic assays are under development, only a few of these can currently be carried out at the point-of-care and, of those that can, none of these are currently able to reliably detect low levels of infection with *S. haematobium* with high specificity [28,30,31,32]. Alternatively, molecular diagnosis using polymerase chain reaction (PCR) or quantitative PCR (qPCR) to detect and amplify *S. haematobium*-specific DNA within urine samples has been shown to be extremely sensitive and specific [33,34,35,36,37,38,39]. PCR assays, and crucial preliminary steps needed to isolate DNA from urine samples, however, require expensive and fragile technical equipment, suitable laboratory infrastructure and trained laboratory personnel—all rarely available within schistosomiasis-endemic areas, thus impeding the use of these methods at the point-of-care [40,41,42]. For these reasons, a variety of alternative and portable DNA amplification technologies have been developed for diagnostic purposes, such as loop-mediated isothermal amplification (LAMP) and recombinase polymerase amplification (RPA), the latter of which has been used to detect trace levels of *S. haematobium* ova-derived DNA within laboratory-prepared samples as well as in clinical urine samples [43,44,45,46]. Unlike PCR, the RPA assay uses a low-temperature isothermal reaction, requires only minimal equipment and takes comparatively far less time to carry out [34,40]. In addition, the RPA can be performed using lyophilised reagents that do not require a cold chain and also by using a crude DNA extraction process that can be easily prepared under field conditions, rendering the RPA well suited for point-of-care use within schistosomiasis-endemic areas [46].

Here, we assessed the analytical and clinical diagnostic performance of a previously developed and field-deployable RPA assay (RT-ShDra1-RPA) that targets the *S. haematobium* Dra1 repeat genomic region [46]. This was carried out using varying concentrations of commercially synthesised *S. haematobium* Dra1 copies as well as laboratory-prepared ddH_2_O samples spiked with differing numbers of *S. haematobium* eggs. In addition, we compare the diagnostic performance of the RPA with that of urine-egg microscopy, considered here as an imperfect reference standard diagnostic.

## 2. Results

### 2.1. RT-ShDra1-RPA Analytical Sensitivity

#### 2.1.1. Synthesised ShDra1 Copies

**Figure 1:**
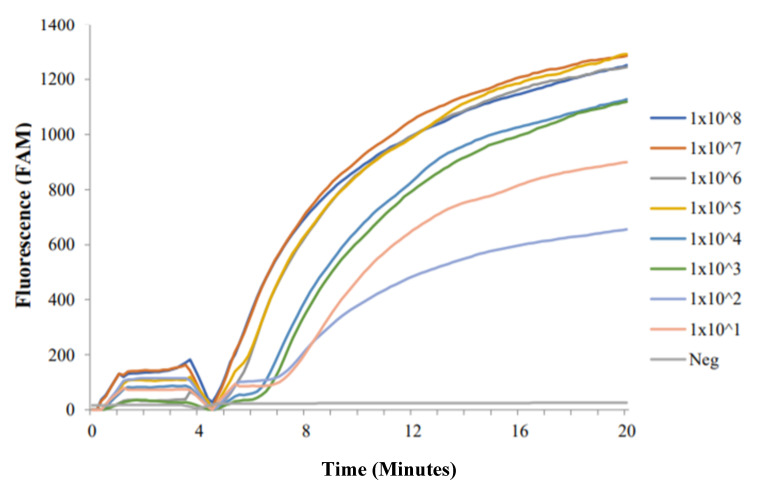
Commercially synthesised Dra1 fragment amplification profiles using RT-ShDra1-RPA assay. All dilution concentrations were successfully detected and amplified. The negative ddH_2_O control (‘Neg’) did not amplify. A temporary reduction in fluorescence was seen at minutes 4–5 as samples were removed to resuspend reagents and then promptly returned to the testing device.

All concentrations of synthetic Dra1 DNA resulted in positive RPA amplification curves (Figure 1). The RT-ShDra1-RPA assay was able to detect as little as 1 × 10^1^ copies of commercially synthesised Dra1 DNA. It was also observed that, in lower concentrations (1 × 10^4^, 1 × 10^3^, 1 × 10^2^ and 1 × 10^1^), the time taken for the onset of fluorescence amplification was increased by ~1–2 min and the final fluorescence level reached was lower than that of higher concentrations (1 × 10^5^ and above). Although the overall fluorescence level was higher after 20 min for 1 × 10^1^ synthesised Dra1 DNA than for 1 × 10^2^ synthesised Dra1 DNA, the time taken for 1 × 10^1^ synthesised Dra1 DNA to amplify and fluoresce was longer than that for 1 × 10^2^ synthesised Dra1 DNA (~30 s). A temporary reduction in fluorescence was seen at minutes 4–5 as samples were removed to resuspend reagents and then promptly returned to the testing device.

#### 2.1.2. *S. haematobium* Eggs: Laboratory-Spiked Samples

**Figure 2:**
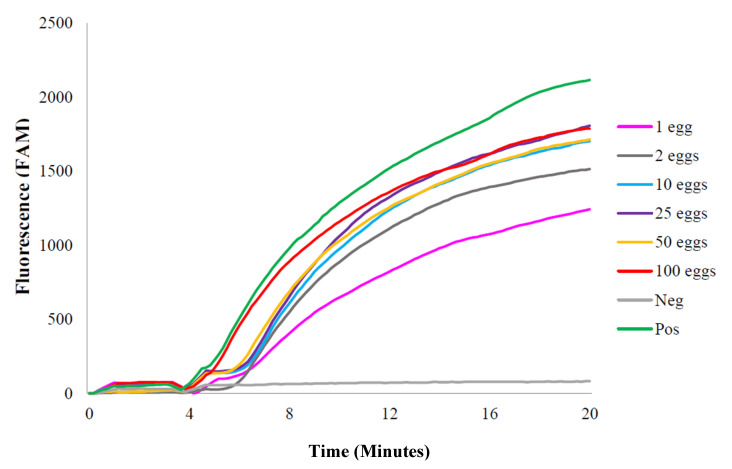
Laboratory-prepared *Schistosoma haematobium* ova-spiked sample plots using RT-ShDra1-RPA assay. All quantities of laboratory-prepared *S. haematobium* ova-spiked samples were successfully detected and amplified. The positive DNA control (‘Pos’) was also detected and amplified. The negative dd H_2_O control (‘Neg’) did not amplify. A temporary reduction in fluorescence was seen at minutes 4–5 as samples were removed to resuspend reagents and then promptly returned to the testing device.

All groups with different quantities of *S. haematobium* egg-spiked samples resulted in positive RPA curves (Figure 2). The RT-ShDra1-RPA assay was able to detect DNA from as little as one egg per sample. For very low egg counts (1 or 2 eggs), the time taken for the onset of fluorescence amplification was again increased by ~1–2 min and the final fluorescence level reached was lower than that of higher egg concentrations (>10 eggs). A temporary reduction in fluorescence was seen at minutes 4–5 as samples were removed to resuspend reagents and then promptly returned to the testing device.

### 2.2. RT-ShDra1-RPA Clinical Performance

Clinical diagnostic performance of the RT-ShDra1-RPA assay compared with urine-egg microscopy is detailed in Table 1.

Among all egg-positive samples (*n* = 158), 10 were negative by RPA (false-negative). Among all egg-negative samples (*n* = 10), none were false-positive by RPA. Overall sensitivity and specificity values were therefore 93.7% (±88.7–96.9) and 100% (±69.1–96.9), respectively. Positive and negative predictive values (PPV/NPV) were 100 (±97.5–100) and 50 (±27.2–72.8), respectively. Three of 12 non-template ddH_2_O samples that were randomly incorporated into testing were false-positive by RPA.

RT-ShDra1-RPA sensitivity and negative predictive values relative to degree of urine-egg output are described in Table 2.

Notably, 42% of samples were classed as ultra-low (1–9 eggs/mL urine), 31% of samples were classed as low (10–49 eggs/10 mL urine) and 73% of samples were classed as either low or ultra-low. Relatively few samples (5%) were classed as either ultra-high (>400 eggs/10 mL urine) or high (16%) egg output (50–399 eggs/10 mL urine) and 6% of urine samples were classed as urine-egg-negative.

The RT-ShDra1-RPA assay correctly identified 64 of all 70 (91.4%) urine-egg-positive results categorised as ultra-low. When ultra-low and low egg output categories were amalgamated, the RT-ShDra1-RPA correctly identified 113 of 122 (92.6%) samples positive by urine-egg microscopy. In addition, the RT-ShDra1-RPA correctly identified 15 of the 16 (93.8%) samples that had an egg output average of just ≤1 egg per/10 mL urine as positive.

Notably, 6 of the 70 samples deemed positive by urine-egg microscopy and categorised as ultra-low egg output were false-negative using RT-ShDra1-RPA, resulting in a sensitivity value of 91.4% (±82.2–96.8). When amalgamating ultra-low and low egg count categories, nine of the 122 egg-positive samples were false-negative by RT-ShDra1-RPA, giving a sensitivity value of 92.6% (±86.5–96.6). Because false-positive and true-negative results have only a binary outcome (positive or negative) and are not associated with a urine-egg count value, specificity values remained constant across all egg count categories (100 ± 69.2–100). Because positive predictive values relative to urine-egg output were influenced only by the number of true-positive samples within a given egg count category, rather than diagnostic agreement between urine-egg microscopy and RT-ShDra1-RPA, those results are not reported.

RT-ShDra1-RPA negative predictive value was notably reduced as egg output declined. As there were six false-negatives by RT-ShDra1-RPA categorised as ultra-low egg output, and because 10 samples were deemed true-negative, the negative predictive value was 62.5% (±35.4–84.8) for ultra-low egg output. When amalgamating ultra-low and low egg count categories (nine false-negative results and 10 true-negative results by RT-ShDra1-RPA), a negative predictive value of 52.6% (± 28.9–75.6) was given. By comparison, only one false-negative result was given for those 27 egg-positive samples categorised as high output (negative predictive value: 90.9% (±58.7–99.8)), and no false-negatives were given for those nine egg-positive samples categorised as ultra-high output (negative predictive value: 100% (±69.2–100)).

## 3. Discussion

Accurate diagnosis of urogenital schistosomiasis is vital for ongoing disease surveillance and control, particularly when aiming to achieve and sustain elimination within endemic areas. Through MDA with praziquantel, a marked reduction in the transmission of schistosomes and schistosomiasis-associated morbidity has been achieved throughout many areas of sub-Saharan Africa by reducing human transurinal excretion of *S. haematobium* eggs. This has, however, caused great difficulties in reliably diagnosing patients with low levels of infection when using standard diagnostic methods such as urine-egg microscopy, highlighting the urgent need for rapid and highly sensitive diagnostic tools that can be used at the point-of-care to promptly identify infected individuals.

The RT-ShDra1-RPA assay successfuly detected and amplified as little as 1 × 10^1^ copies of commercially synthesised Dra1 DNA as well as DNA from as little as one *S. haematobium* egg per 100 µL ddH_2_O sample, showing that the assay is capable of detecting minute levels of DNA. These results also show that the crude and field-deployable Qiagen SpeedXtract Nucleic Acid Kit (Qiagen, Germany) used to extract DNA from egg-spiked samples, is efficient at extracting very low quantities of DNA (i.e., one single egg).

The ability of the RT-ShDra1-RPA assay to detect low levels of *S. haematobium* DNA was additionally demonstrated when used to assess urine samples from an *S. haematobium* endemic elimination setting (Zanzibar). Sensitivity of the RT-ShDra1-RPA assay was high (91.4% ±82.2–96.8), even when assessing only those samples deemed to have ultra-low egg output by urine-egg microscopy (1–9 eggs/mL urine). In addition, as 93.8% of samples that had an egg output average of just ≤1 egg per/10 mL urine were successfully detected by RT-ShDra1-RPA. These data show that the RT-ShDra1-RPA is able to detect low levels of infection within infected individuals reliably. It is pertinent to note that diagnosis by urine-egg microscopy was performed by two highly skilled technicians and requires a great deal of training, experience and time to carry out effectively. As such, the ability of the RT-ShDra1-RPA assay, which requires comparatively less training, experience and time to perform, to also reliably detect low levels of infection that can be easily missed by microscopy is an important finding [39].

As egg output declined, the negative predictive value of the RT-ShDra1-RPA assay was notably reduced. As an example, for ultra-low egg counts, the negative predictive value was only 62.5% (±35.4–84.8). This result, however, is heavily influenced by the small sample size of urine samples false-negative by RT-ShDra1-RPA (*n* = 6) and true-negative by RT-ShDra1-RPA (*n* = 10). As such, given this small sample size (*n* = 16), the resulting negative predictive value should be considered with caution. The negative predictive value was further reduced when combining low and ultra-low egg output categories (52.6% ± 28.9–75.6). Again, however, this is because the number of samples deemed true-negative by RT-ShDra1-RPA remained constant (*n* = 10), whereas the number of false-negative samples increased only marginally (*n* = 9). Because of the low sample size (*n* = 19), this result should also be considered with caution.

### Study Limitations and Future Work

The primary limitation of this study is that qPCR, a highly sensitive molecular diagnostic assay, was not used as the reference standard. qPCR was not used here because the Speedxtract DNA isolation method has not been validated for use with qPCR and because of limited financial and project resources. However, these comparisons should be made in further investigations and to support development of the methodology. Comparing the diagnostic performance of the RT-ShDra1-RPA assay and urine-egg microscopy with a qPCR reference standard would allow assessment as to whether the RT-ShDra1-RPA assay is more sensitive than urine-egg microscopy as infections missed by microscopy but detected by qPCR should also be detected by RT-ShDra1-RPA. Extracting DNA from urine samples using an additional method of DNA extraction applicable for use with qPCR, and carrying out qPCR analysis on all samples, would also allow the concentration of *S. haematobium* DNA within samples to be quantified as this is not possible using RT-ShDra1-RPA. In doing so, the ability of the RT-ShDra1-RPA to detect minute levels of *S. haematobium* DNA within urine sample extracts could be more thoroughly assessed. Although not able to fully quantify the amount of DNA, the RT-ShDra1-RPA assay may be of use as a semi-quantitative measure of DNA concentrations in samples through evaluation of the time to fluorescence amplification as, here, it is suggested that the onset of amplification is earlier in samples containing higher levels of DNA. This, however, requires further investigation to clarify.

Another limitation of the study is the low proportion of egg-negative samples to egg-positive results determined by microscopy. More egg-negative samples would have allowed for a more thorough analysis and understanding of RT-ShDra1-RPA negative predictive values. Moreover, assessing a higher volume of egg-negative samples would provide an opportunity to more thoroughly assess the ability of RT-ShDra1-RPA to detect infection within samples deemed egg-negative by urine-egg microscopy, particularly if using qPCR as the reference standard. Improved sensitivity in this way is a crucial requirement of any future diagnostic assay. Alternative methods of molecular diagnosis, such as qPCR, are capable of doing this as a positive diagnosis relies only on the detection of *S. haematobium* DNA, rather than the detection of eggs that can easily be missed by microscopy [39,40]. As such, any future assessment of the RT-ShDra1-RPA should incorporate a higher volume of urine samples negative by urine microscopy and all microscopy and RT-ShDra1-RPA results should be compared to a qPCR reference standard.

Of the 12 negative non-template ddH_2_O samples randomly integrated into sample analysis, three were positive by RT-ShDra1-RPA, demonstrating the detection and amplification of contaminant DNA within these samples, which most likely occurred during sample or reaction preparation. To ensure optimum RT-ShDra1-RPA specificity, care should be taken when preparing samples to avoid any DNA contamination and hence to avoid false-positive results. In addition, for further quality control and quality assurance, an internal housekeeping gene RPA control should be developed and assessed in order to ensure that all RT-ShDra1-RPA-possitive outcomes are not a result of assay or sample preparation faults.

Whilst DNA extraction took place directly at the Public Health Laboratory Ivo de Carneri (PHL-IdC) in Pemba using the SpeedXtract Nucleic Acid Kit (Qiagen, Germany), RPA analysis using the AmpliFire isothermal nucleic acid testing device (Douglas Scientific, Alexandria, MN, USA) was performed in the laboratories of the Natural History Museum (London, UK). Future assessment of the RT-ShDra1-RPA assay in African laboratories as well as at the peripheral level (directly in schools, health facilities or at mobile laboratories) to fully evaluate its potential as a portable and robust diagnostic assay, suitable for use at the point-of-care in schistosomiasis endemic areas, should be carried out [47].

To conserve resources, a volume of 50 µL urine was used for DNA extractions using the SpeedXtract Nucleic Acid Kit (Qiagen, Germany). Increasing this volume—for example, to 100 µL—may increase RT-ShDra1-RPA sensitivity as *S. haematobium* DNA may then be detected in those nine samples negative by RT-ShDra1-RPA that were positive by egg microscopy and deemed to have low and ultra-low egg output (mean egg output within these nine samples was 6.8 eggs/mL urine). This too requires further investigation to clarify.

This study used standard protocols as outlined by the various materials’ manufacturers’ instructions. There is, however, scope for more tailored and refined sample preparation and assay running methodologies to be developed and trialled to further improve RT-ShDra1-RPA performance in endemic settings. In addition, multiple comparisons between the RT-ShDra1-RPA and other methodologies used to diagnose urogenital schistosomiasis, such as qPCR, urine-circulating cathodic antigen (CCA) or urine-circulating anodic antigen (CAA) lateral-flow test strips, or the highly-sensitive UCP-LF-CAA assay, especially in low-prevalence settings, should be carried out to help determine the optimal method of diagnosis [25,30,41].

## 4. Materials and Methods

### 4.1. RPA Reactions

RT-ShDra1-RPA assay design (primers/probes and preliminary testing) has been described in detail elsewhere [46]. RPA reactions were performed using TwistDx (Cambridge, UK) RPA Exo Kits according to the manufacturer’s instructions. All reactions were carried out within the laboratories of the Natural History Museum (London, UK) using the AmpliFire isothermal nucleic acid testing device (Douglas Scientific, Alexandria, MN, USA). This is a robust, lightweight, hand-held and battery-powered instrument that can be easily navigated using a touchscreen interface. Assay settings can be quickly uploaded by use of a printed QR code and integrated barcode scanner and assay results can be visualised using the device’s touchscreen and exported using an integrated and external storage compatible USB port, omitting the need for sophisticated and fragile readout equipment. Reactions contained 2.1 µL of each primer (forward and reverse; 10 pmol), 0.6 µL of the internal probe (10 pmol), 29.5 µL rehydration buffer, 2.5 µL (280 nM) magnesium acetate (MgAc) and 5 µL template DNA. Total reaction volume was made up to 50 µL by adding 8.2 µL ddH_2_O.

Reactions were carried out by preparing a reagent master mix containing forward and reverse primers, internal probe, rehydration buffer and ddH_2_O. A total of 42.5 µL master mix was added to each lyophilised RPA pellet, followed by 5 µL template DNA. Next, 2.5 µL MgAc was pipetted into tube lids and the lids were carefully closed so that the MgAc was not introduced into the reaction mix prematurely. Reaction tubes were inverted by hand to mix reagents and to initiate assay reactions upon introduction of MgAc. Tubes were then promptly centrifuged and placed into position within the AmpliFire testing device. The pre-programmed assay conditions that run at a constant isothermal temperature of 42 °C and are programmed by scanning a printed QR code using the integrated barcode scanner were then initiated.

After 4 min, tubes were temporarily removed from the AmpliFire testing device, inverted by hand to resuspend reagents, centrifuged and promptly returned to device to continue amplification. One reaction cycle takes 20 min in total. Samples were considered positive if amplification curves followed a smooth, sigmoid profile and amplified beyond a threshold marker, as estimated based on background flourescence detected during the initialisation period (0–4 min). Samples were considered negative if amplification curves did not cross this threshold marker.

### 4.2. RT-ShDra1-RPA Analytical Sensitivity

#### 4.2.1. Synthesised ShDra1 Copies

The analytical sensitivity of the RT-ShDra1-RPA assay was previously determined at 1 fg of DNA using *S. haematobium* gDNA [46]. Here, we further evaluated the assay’s analytical sensitivity with regard to its lower limit of detection (LoD) in relation to DNA copies. Synthetic ShDra1 DNA (502 bp) was commercially synthesized (GeneArt, Invitrogen, Waltham, MA, USA), and a 1 ng/µL stock solution was provided containing 18 × 10^8^ copies/µL. This stock solution was then diluted to a working concentration of 1 × 10^8^ copies/µL and serial dilutions were then prepared down to 1 × 10^1^ copies/µL. Each 10-fold dilution was tested, in duplicate, using the RT-ShDra1-RPA as described above and using 1 µL of the synthetic DNA dilution. Positive gDNA and negative ddH_2_O (non-template) controls were included. Positive control *S. haematobium* gDNA was obtained from the Schistosomiasis Collection at the Natural History Museum (SCAN) repository and originated from a Senegalese *S. haematobium* strain [46,48].

#### 4.2.2. *S. haematobium* Eggs: Laboratory-Prepared Samples

To further assess its LoD in relation to numbers of eggs, the RT-ShDra1-RPA was tested using known numbers of frozen *S. haematobium* eggs. Purified *S. haematobium* eggs (Egyptian strain), provided in frozen solution, were obtained from the Schistosomiasis Resource Centre (Biomedical Research Institute, San Antonio, TX, USA). Using a dissecting microscope, individual eggs were captured using a micro-pipette and transferred to a new sample tube. Groups of eggs of different quantities (1, 2, 10, 25, 50 and 100 eggs) were made up in 100 µL ddH_2_O. DNA was extracted from each group using the Qiagen SpeedXtract Nucleic Acid Kit (Qiagen, Germany) as described previously [46]. Each group of eggs was tested in duplicate using the RT-ShDra1-RPA assay as outlined above, using 1 µL of the DNA extracts. A positive *S. haematobium* gDNA control and a negative non-template control were included as above.

### 4.3. RT-ShDra1-RPA Clinical Performance

#### 4.3.1. Study Area and Urine Samples

Urine samples were originally provided by school children aged 9–12 years from Pemba island, Zanzibar, as part of the Zanzibar Elimination of Schistosomiasis Transmission (ZEST) project (2011–2017), in 2016 and 2017 [49,50,51]. All urine samples were produced between 10:00 am and 14:00 pm to coincide with optimum egg output [18]. All urine samples had been previously screened for *S. haematobium* infection by urine filtration (10 mL) and egg microscopy during ZEST parasitological surveys according to a standard protocol outlined previously [39,49]. One single urine filtration was performed and each slide was read by two independent and experienced field technicians at the Public Health Laboratory-Ivo de Carneri (PHL-IdC) in Pemba. Urine-egg microscopy was considered positive when at least one egg was detected in at least one reading and considered negative when no eggs were detected within either reading. Average egg count values were calculated and classified into the following egg count categories: ultra-high (400+ eggs/10 mL urine), high (50–399 eggs/10 mL urine), low (10–49 eggs/10 mL urine) and ultra-low (1–9 eggs/10 mL urine). Aliquots of 1.5 mL urine samples were frozen at −20 °C as a resource for further diagnostic assessment.

A total of 168 frozen urine samples were selected for RPA clinical performance analysis, 100 of which were selected based on urine-egg count data so that ultra-high (400+ eggs/10 mL urine), high (50–399 eggs/10 mL urine), low (10–49 eggs/10 mL urine) and ultra-low (1–9 eggs/10 mL urine) were represented within the study. The remaining 68 samples were randomly selected. An additional 12 negative control non-template (ddH_2_O) samples were randomly incorporated into the processing to further assess analytical performance of the RT-ShDra1-RPA. These samples were prepared within the laboratories of the Natural History Museum (London, UK) and were not incorporated into any statistical analyses. All 180 samples were randomised and were run blind to any associated data.

#### 4.3.2. DNA Isolation and RT-ShDra1-RPA Use with Clinical Urine Sample Extracts

DNA isolation from urine samples was performed at the PHL-IdC in Pemba using the SpeedXtract Nucleic Acid Kit (Qiagen, Germany) as described elsewhere [46]. This is a rapid extraction process that can be easily performed in resource-poor field settings. Urine samples previously stored at −20 °C were defrosted/vortexed and a conservative volume of 50 µL was used for DNA extractions. All extracts were tested using the RT-ShDra1-RPA assay as described above, using 5 µL of extract per reaction, within the laboratories of the Natural History Museum (London, UK). The AmpliFire testing device is capable of processing up to eight samples per assay run (20 min), and so a fresh master mix was prepared every other assay run (i.e., each master mix was sufficient for 16 samples). Likewise, one *S. haematobium* egg-derived gDNA positive control (1 µL template gDNA (as above) + 9 µL ddH_2_O) and one negative control (10 µL ddH_2_O) were included for every sixteen samples.

### 4.4. Statistical Analysis

To assess the clinical diagnostic performance of the RT-ShDra1-RPA assay, using urine-egg microscopy results as a reference test, sensitivity, specificity and positive/negative predictive values (PPV/NPV) were calculated using the *epiR* package [52] within R version 1.3.959 [53]. Overall values were calculated using all 168 urine samples. To assess diagnostic performance of the RT-ShDra1-RPA assay in terms of urine-egg output, additional sensitivity and NPV calculations were performed relative to urine-egg count categories (Supplementary Materials).

### 4.5. Ethical Approval and Consent to Participate

The ZEST study protocol was reviewed and obtained ethical approval by (i) the Zanzibar Medical Research Ethical Committee (ZAMREC) in Zanzibar, United Republic of Tanzania (reference no. ZAMREC 0003/Sept/011); (ii) the “Ethikkomission beider Basel” (EKBB) in Switzerland (reference no. EKBB 236/11); and (iii) the institutional review board of the University of Georgia (IRB UGA project no. 2012–10138-0). The trial has been registered at the International Standard Randomised Controlled Trial Number Register (ISRCTN48837681; http://www.controlled-trials.com/ISRCTN48837681). Ethical approval for the analytical and clinical assessment of the RT-ShDra1-RPA assay was also granted by authorisation from the London School of Hygiene and Tropical Medicine (LSHTM) MSc Research Ethics Committee (Ref: 15,612).

Written informed consent from parents or legal guardians of participating children in Zanzibar was obtained for urine collection and storage, including examination for *S. haematobium* infections with newly developed diagnostic techniques at a later point in time. School-aged children were offered praziquantel (40 mg/kg) against schistosomiasis free of charge in the frame of the biannual island-wide mass drug administration campaigns.

## 5. Conclusions

Here, we demonstrate that the rapid, isothermal and field-deployable RT-ShDra1-RPA assay is able to detect and amplify trace levels of *S. haematobium* DNA as well as correctly identify *S. haematobium* infections using clinical urine samples, even when only a very low number of eggs (<10 eggs/10 mL urine) is expelled. Additionally, and importantly, this work was completed using a rapid and crude DNA extraction process that can be carried out under resource-poor endemic field settings.

Although additional development and assessment is required before the upscaled and routine diagnostic use of the RT-ShDra1-RPA, the assay proved highly portable and could perform well as part of a mobile laboratory in resource-poor settings to enable point-of-care molecular diagnosis of urogenital schistosomiasis. As such, with further development, the RT-ShDra1-RPA shows promise for future use as a means of reliably carrying out active surveillance of high-risk groups or test-and-treat/passive surveillance within health facilities to promptly identify those harbouring low levels of infection still able to maintain pathogen transmission. In doing so, the control and elimination of urogenital schistosomiasis transmission can be more effectively achieved, greatly reducing the degree of debilitating disease-associated morbidities experienced by those in endemic areas.

## Figures and Tables

**Table 1:** Diagnostic performance of the RT-ShDra1-RPA assay (index), compared to a urine-egg microscopy reference test*. Sensitivity, specificity, positive predictive value (PPV) and negative predictive value (NPV) ± confidence intervals (±95% CI) are provided. Associated calculations are indicated within brackets.

		Urine-Egg Microscopy * (Reference)			
		+	−	Total	
RT-ShDra1-RPA (Index)	+	148	0	148	PPV % (±95% CI): 100 (97.5–100) (48/148)
	−	10	10	20	NPV % (±95% CI): 50 (27.2–72.8) (10/20)
	Total	158	10	168	
		Sensitivity % (±95% CI): 93.7 (88.7–96.9) (148/158)	Specificity % (±95% CI): 100 (69.1–100) (10/10)		

* All urine samples were subject to urine-egg microscopy readings in duplicate. Urine-egg microscopy was considered positive when at least one egg was detected in at least one reading and considered negative when no eggs were detected within either reading. ‘+’ denotes a positive outcome, ‘−’ denotes a negative outcome.

**Table 2:** All urine samples (*n* = 168) categorised by degree of urine-egg output *. Sensitivity and negative predictive value analysis (±95% CI) of the RT-ShDra1-RPA assay (index), compared to a urine-egg microscopy reference test across all egg count categories—ultra-high, high, low and ultra-low—are also outlined **. Low and ultra-low egg count categories were also amalgamated and analysed. Sensitivity and negative predictive value calculations are indicated within brackets.

Reference	Index	Analysis	Ultra-High (>400 Eggs/10 mL Urine)	High (50–399 Eggs/10 mL Urine)	Low (10–49 Eggs/10 mL Urine)	Ultra-Low (1–9 Eggs/10 mL Urine)	Low and Ultra-Low (1–49 Eggs/10 mL Urine)	Egg-Negative
		Total number of samples within egg count category (% total) *	9 (5%)	27 (16%)	52 (31%)	70 (42%)	122 (73%)	10 (6%)
Urine-egg microscopy *	RT-ShDra1-RPA	Sensitivity % (±95% CI) **	100 [9/9] (66.4–1)	96.3 [26/27] (81–99.9)	94.2 [49/52] (84.1–98.8)	91.4 [64/70] (82.2–96.8)	92.6 [113/122] (86.5–96.6)	NA
		Negative Predictive Value % (±95% CI) **	100 [10/10] (69.2–100)	90.9 [10/11] (58.7–99.8)	76.9 [10/13] (64.2–95)	62.5 [10/16] (35.4–84.8)	52.6 [10/19] (28.9–75.6)	NA

* All urine samples were subjected to urine-egg microscopy in duplicate and mean urine-egg output was calculated. Urine-egg microscopy was considered positive when at least one egg was detected in at least one reading and considered negative when no eggs were detected within either reading. Egg count values are mean values taken from two independent microscopic assessments of one urine sample. ** Sensitivity and NPV analysis were carried out using total number of true positives and false negatives within that egg count category, total false positives and total true negatives. Only true-positive and false-negative outcomes have an associated urine-egg count value. Sensitivity and specificity testing cannot be carried out on egg-negative samples and so ‘NA’ is given.

## Supplementary Materials

The supplementary materials are available online.

## Abbreviations

bp	Base pairs
CAA	Circulating anodic antigen
CCA	Circulating cathodic antigen
FAM	6-carboxyflourescein
fg	Femtogram
gDNA	Genomic DNA
LoD	Limit of detection
LSHTM	London School of Hygiene and Tropical Medicine
MDA	Mass drug administration
NHM	Natural History Museum, London, UK
NPV	Negative predictive value
NTD	Neglected tropical disease
PC	Preventative chemotherapy
PCR	Polymerase chain reaction
PHL-IdC	Public Health Laboratory Ivo de Carneri
PPV	Positive predictive value
qPCR	Quantitative/real-time PCR
RPA	Recombinase polymerase amplification
Sh	*Schistosoma haematobium*
STI	Sexually transmitted infection
Th2	T-helper type 2
